# Test conditions can significantly affect the results of in vitro cytotoxicity testing of degradable metallic biomaterials

**DOI:** 10.1038/s41598-021-85019-6

**Published:** 2021-03-23

**Authors:** Eva Jablonská, Jiří Kubásek, Dalibor Vojtěch, Tomáš Ruml, Jan Lipov

**Affiliations:** 1 Department of Biochemistry and Microbiology, University of Chemistry and Technology, Prague, Technická 5, 166 28, Prague 6, Czech Republic; 2 Department of Metals and Corrosion Engineering, University of Chemistry and Technology, Prague, Technická 5, 166 28, Prague 6, Czech Republic

**Keywords:** Cell culture, Biomaterials

## Abstract

In vitro cytotoxicity testing is an indispensable part of the development of new biomaterials. However, the standard ISO 10993-5 enables variability in the testing conditions, which makes the results of the test incomparable. We studied the influence of media composition on the results of the cytotoxicity test. Solutions of ZnCl_2_ served as simulated extracts and we also used extracts of three types of Zn-based and Mg-based degradable metals. We incubated the cells with the solutions prepared in two types of media with two concentrations of serum (5 and 10%). We compared the toxic effect of the extracts on L929 murine fibroblast-derived cell line, which is recommended by ISO standard and on “osteoblast-like cells” U-2 OS. We also compared two methods of exposition: solutions were added either to a sub-confluent layer or to the cell suspension. We evaluated the metabolic activity of the cells using the resazurin test. We found out that in vitro cytotoxicity is dramatically influenced by the concentration of serum and by the type of the medium as well as by the type of exposition and type of cells. Therefore, when performing in vitro cytotoxicity testing of biomaterials, the authors should carefully specify the conditions of the test and comparison of different studies should be carried out with caution.

## Introduction

In vitro methods cannot capture all complexities in the body during in vivo tests. Nevertheless, they are an indispensable part of the evaluation process of medical devices including implants. They precede in vivo tests because they are a valuable indicator of the potential behaviour of the newly developed biomaterial in contact with the tissue in vivo^1^. Standardized tests for biological safety evaluation are described in ISO 10993 (Biological evaluation of medical devices). The first choice is the test for cytotoxicity described in part ISO 10993-5: 2009. Nevertheless, this standard is only a set of recommendations and many parameters and conditions can be adjusted, if properly justified.

The in vitro test shall be performed either on the tested sample itself (direct contact test or indirect contact test, i.e., agar diffusion test), or on an extract of the tested sample (so-called elution test), the latter mentioned being the most frequently used. The more detailed description can be found in^2^. Briefly, during the elution test, the tested materials are immersed into the medium and the cells are then exposed to the resulting extracts. Cell viability after the exposition is evaluated and compared to the unaffected control. This test seems to be the most appropriate method of exposure because it can be used for a very broad spectrum of types of materials of different shapes. There is no risk of cellular trauma as in the case of direct contact of the material with the cell layer and there is no risk of thermal shock as in the case of the agar diffusion method^3^. However, the preparation of extracts and other conditions of the test as described in the ISO standard, part 5 and part 12, are not strictly defined. The steps and options are summarized in Table 1. Thus, this latitude can lead to major discrepancies between the results obtained by different laboratories.

**Table 1:** Parameters and options of elution test stated in ISO 10993.

Parameter	Options	Citation
Extraction volume	125 mm^2^ · ml^−1^ Or 0.2 g · ml^−1^	ISO 10993-12
The velocity of orbital shaking during extraction	Shaking required, but velocity not specified	ISO 10993-12
FBS concentration in extraction medium	5%, 10% or without FBS	ISO 10993-5 Annex A
Cultivation medium composition (type)	According to the cell type	ISO 10993-5
Time of extraction	24 h^a^	ISO 10993-5
Cell type	e.g. L929 cells	ISO 10993-5 Annex C
Mode of exposition	Cell layer or freshly suspended cells	ISO 10993-5
Incubation time	At least 24 h	ISO 10993-5

^a^When cultivation medium with serum is used.

Originally, biomaterials of the first generation were bioinert, i.e., the release of the substances from the material was undesirable. The ISO standard describes so-called exaggerated conditions. Nowadays, degradable metallic biomaterials for temporary implants attract great attention (reviewed in^4^). In this case, the degradation is expected and required and some conditions for in vitro testing stated in the standard might be too stringent leading to nonrelevant results. There are attempts to bring in vitro testing of degradable metallic biomaterials closer to physiological conditions. In the following part, parameters of the test will be described, taking the testing of degradable metals into account.

1. Extraction volume is one of the most important parameters. Extraction surface-to-volume or mass-to-volume ratio recommended by ISO 10993-12 is 125 mm^2^ · ml^−1^ or 0.2 g · ml^−1^, respectively. However, for degradable materials, these ratios are inappropriate. It has been shown that for obtaining relevant in vitro cytotoxicity results with magnesium-based materials, it is necessary to use up to 10 times more extraction medium than recommended in the standard. This provides that buffering capacity and osmolarity will not be severely altered and ensures conditions better mimicking those in vivo with body fluid circulation and removal of the degradation products from the site of implantation^5,6^.
2. Extraction of the tested materials should be performed at 37 °C on an orbital shaker. Shaking eliminates the concentration gradient in the vicinity of the sample. It could, to a certain extend, mimic the circulation of body fluids in vivo. The velocity, however, is not specified. This could also lead to discrepancies. Vigorous shaking can lead to excessive abrasion of the material and increase the corrosion rate. If there are more pieces of the material, it is also reasonable to extract those pieces in separated vessels to prevent abrasion, which is another factor not specified in the ISO standard.
3. Another vaguely stated but crucial parameter which could cause major discrepancies is the concentration of foetal bovine serum (FBS) in the medium. FBS represents the blood depleted of cells and coagulation factors. It contains amino acids, proteins, vitamins, carbohydrates, lipids, hormones, growth factors, inorganic salts, trace elements, and other compounds^7^. The addition of 10% of the FBS to the cultivation medium is a common practice for cell cultivation in vitro.

The ISO standard recommends a cultivation medium supplemented with serum as an extraction medium because FBS enables the extraction of both polar and nonpolar compounds. Extraction in cultivation medium without serum can be used for preferential extraction of polar substances. The standard, however, does not specify subsequent procedure. Probably, the serum should be added after extraction for the successful cell cultivation.

FBS concentration during extraction and during cytotoxicity testing of degradable metallic biomaterials is frequently discussed. First, FBS concentration influences ion release during the extraction. The degradation rate of Mg-based biomaterials in medium was observed to be lower in the presence of FBS^8–11^ and  the degradation rate of Zn-based biomaterials was higher in pure FBS than in medium with 10% FBS^12^. Second, FBS concentration thereafter affects the cytotoxicity of the extracts towards the cells. In the ISO standard (informative Annex A), it is recommended to use exaggerated conditions, i.e., to use the decreased concentration of FBS to 5%, because the proteins of FBS can bind the extracted substances and thus mask their toxic effect.

**Figure 1:**
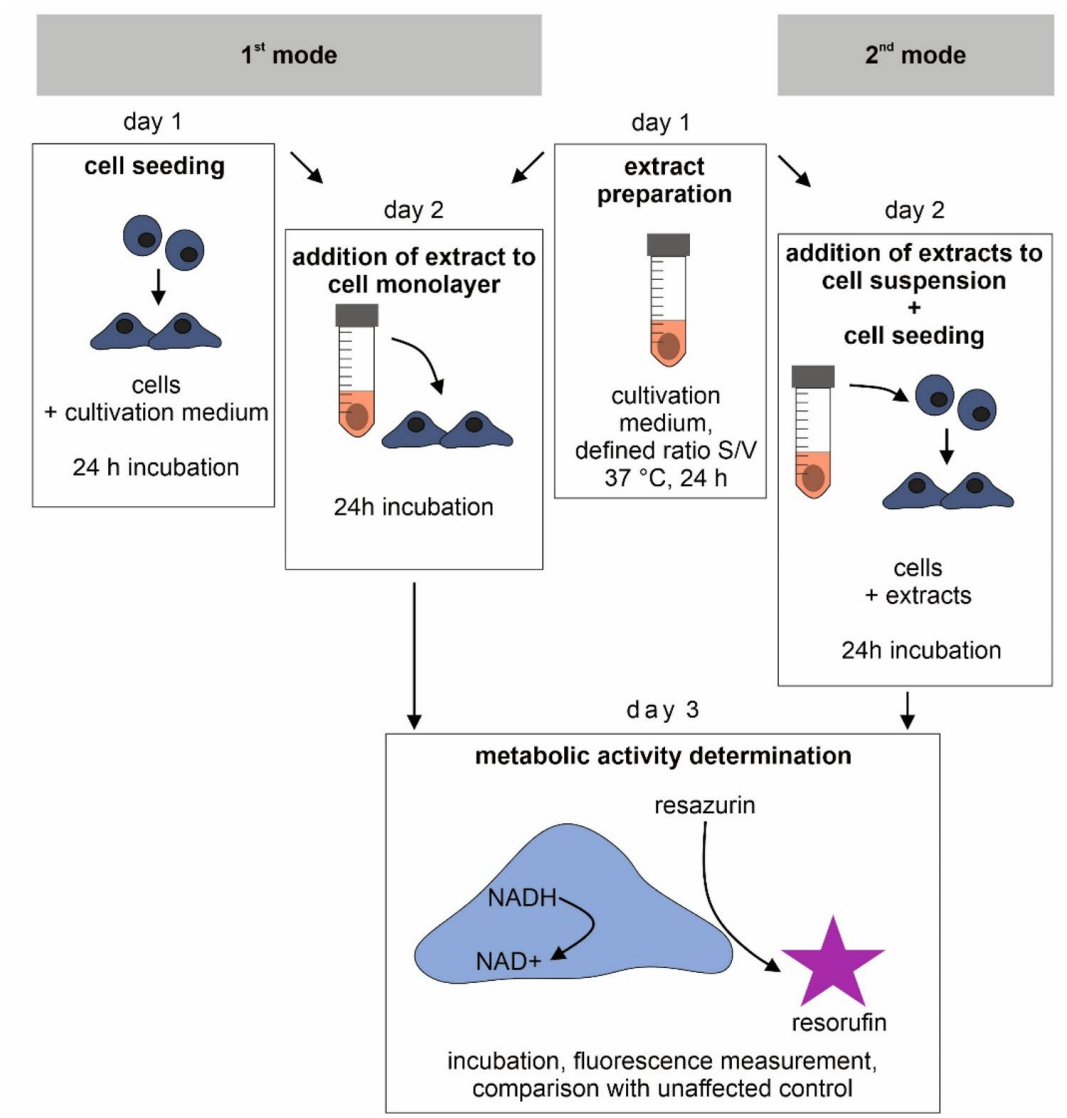
Two modes of exposition. Extracts were either added to a sub-confluent cell monolayer 24 h post-seeding (1st mode) or were added to freshly suspended cells (2nd mode). The figure was created using CorelDRAW 2019, version 21.3.0.755.

(4) The type of cultivation medium is usually dictated by the cell type used, as recommended by the cell depositor. There is a vast number of cultivation media which differ in the constitution and concentration of the components^7^. The variation in the composition of an extraction medium can have a pronounced influence on the corrosion rate as well as on the cytotoxicity results^13^. Besides, many authors do not specify the medium, e.g., some claim only “DMEM medium”, without further specification of glucose concentration or additional buffer content.
(5) According to the standard, it is possible to test the extracts on a sub-confluent cell layer prepared one day before addition. Alternatively, extracts can be added to freshly suspended cells (Fig. 1). This could also influence the results of cytotoxicity testing.

Many authors only claim that “In vitro cytotoxicity testing was performed according to ISO 10993-5 standard using indirect in vitro cytotoxicity tests” with no further specification. Here we use ZnCl_2_ solutions and three examples of degradable metallic materials to show how profound effect on the results of the cytotoxicity test may the conditions have.

## Materials and methods

### Preparation of the simulated extracts

10 mM stock solution of ZnCl_2_ was prepared in dH_2_O and sterilised by filtration. Before the experiment, fresh working solutions (40, 80, 120, and 180 µM) were prepared in each type of cultivation medium used.

### Preparation of the materials

In the present study, three different materials (Table 2) were used for cytotoxicity tests. The selection of these materials reflects their perspective mechanical and corrosion properties regarding applications like biodegradable medical devices^14–16^. Mg_HF_SPS sample was prepared by chemical treatment of magnesium powder in hydrofluoric acid (HF) for 24 h, cold pressing of this powder into a green compact, and subsequent spark plasma sintering at 500 °C. Details of the procedure are given in^14^. Zn_SPS sample was prepared by cold pressing of pure Zn powder into a green compact and subsequent spark plasma sintering at 300 °C with details of the processing in^15^. Zn-0.8 Mg was prepared by the general casting process and subsequent extrusion at 300 °C at a rate of 5 mm/min and extrusion ratio equal to 10, which is in more details described in^16^.

**Table 2:** Materials used for testing.

Designation	Input material	Chemical treatment of powder	Processing
Mg_HF_SPS	Mg powder	HF (24 h)	Cold pressing and SPS (500 °C)
Zn_SPS	Zn powder	–	Cold pressing and SPS (300 °C)
Zn0.8 Mg	Zn, Mg ingots	–	Casting (on air) + extrusion (300 °C and extrusion ratio 10)

Cylindrical samples (20 mm in diameter and 2 mm in height) of Mg_HF_SPS and cuboidal samples (12 × 12 × 3 mm) of Zn_SPS and cylindrical samples (5.5 mm in diameter and 5 mm in height) of Zn0.8 Mg were ground (SiC paper, up to P4000), cleaned and sterilised by immersing into 70% ethanol (2 h) and by subsequent UV exposition (2 h).

### Preparation of the extracts

The prepared samples were transferred to MEM cultivation medium with 5% or 10% fetal bovine serum (FBS) and agitated (130 RPM) at 37 °C in closed vessels for 24 h. The surface to volume ratio was 87.5 mm^2^ · ml^−1^ for all samples. Three triplicates were used for each sample. The extracts were further used for indirect in vitro cytotoxicity tests (undiluted, i.e., 100% and diluted. i.e., 50% and 25% extracts were used) and for ICP-MS measurement.

### Cell culture conditions

L929 cell line derived from murine fibroblasts (ATCC CCL-1™) and U-2 OS cell line derived from human osteosarcoma (ATCC HTB-96™) were maintained in MEM (Minimal Essential Medium, Sigma, M0446) and DMEM (Dulbecco’s Modified Eagle’s Medium, Sigma, D0819), respectively with 10% FBS at standard conditions of 37 °C, 5% CO_2_ and 100% relative humidity. Cells were used from the 3rd passage after thawing and only till the 20th passage. Cells were passaged regularly using trypsin–EDTA solution (Sigma, T4049) which was stopped by addition of the medium with FBS with no subsequent centrifugation.

### Exposition to the extracts using the sub-confluent layer (Fig. 1, 1st mode)

One day before the exposition, L929 cells (or U-2 OS cells) were trypsinized and resuspended in MEM + 10% FBS to create a suspension with a concentration of 1 × 10^5^ cells per ml. Thereafter, 100 µl of the cell suspension was seeded into a 96-well plate, which means the seeding density of 1 × 10^4^ cells per well. After 1 day, the medium was replaced by the ZnCl_2_ solutions prepared as described in “Preparation of the simulated extracts” section or by the extracts prepared as described in “Preparation of the extracts” section. Sole MEM with the corresponding concentration of FBS served as a control. All samples including the control were measured in six parallel replicates. We present the variance of the results also for the control. The 100% value represents the mean value of these control measurements.

### Exposition to the extracts using the cell suspension (Fig. 1, 2nd mode)

At the day of the exposition, L929 cells were trypsinized and resuspended in MEM + 10% FBS to create a suspension with a concentration of 10 × 10^5^ cells per ml. The suspension was then mixed with ZnCl_2_ solutions prepared as described in “Preparation of the extracts” section or by the extracts prepared as described in “Preparation of the extracts” section in 1:9 ratio (1 part of the cell suspension plus 9 parts of the extracts) and 100 µl of the mixture was seeded into 96-well plate in hexaplicates, which means the density of 1 × 10^4^ cells per well. Sole MEM with the corresponding concentration of FBS mixed with the suspension served as a control. All samples including the control were measured in six parallel replicates. We present the variance of the results also for the control. The 100% value represents the mean value of these control measurements.

### Evaluation of metabolic activity

After one day of incubation with the extracts, cell metabolic activity was evaluated using resazurin assay (Fig. 1, day 3)^17^. Cells were washed by phosphate buffer saline (PBS) and resazurin solution (final concentration 25 µg · ml^-1^) in MEM + 10% FBS without phenol red was added. After 1 h of incubation, fluorescence at 560/590 nm (excitation/emission) was measured. Cytotoxicity of the extracts was depicted as a percentage of metabolic activity of the control, i.e., as relative metabolic activity (RMA). First, blank measurement (no cells) was subtracted from all values. The formula used was RMA = (F_sample_)/(F_control_), where F_sample_ stands for the average fluorescence of resorufin produced by the affected cells (i.e. cells incubated with extracts) and F_control_ stands for the average fluorescence of resorufin produced by of the unaffected cells (i.e. control cells). Extracts causing the decrease below 70% of the activity of the control were considered cytotoxic, as described in the standard ISO 10993-5.

### Statistical analysis

**Figure 2:**
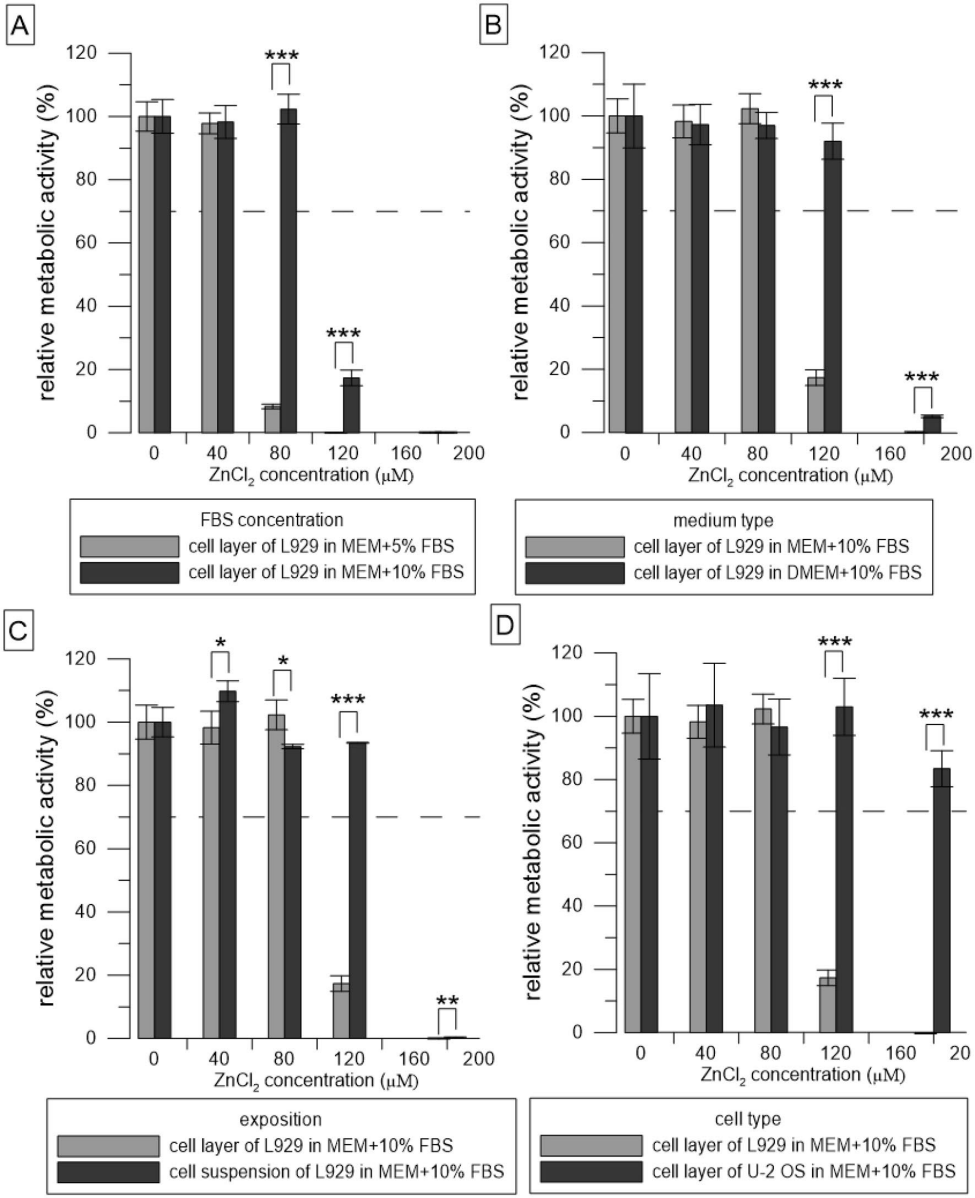
Relative metabolic activity of the cells (resazurin assay) after 24 h incubation with solutions of ZnCl_2_. Metabolic activity of the medium without ZnCl_2_ was taken as 100%. (**A**–**D**) represent the different conditions used: (**A**) compares two concentrations of FBS, (**B**) compares two types of media, (**C**) compares two ways of exposition, and (**D**) compares two cell types. The dashed line stands for the normative limit of 70% metabolic activity of the control. Error bars stand for the standard error deviation of six replicates. Significance codes according to p value: 0 ‘***’0.001 ‘**’0.01 ‘*’0.05 (two-sample t-test).

**Figure 3:**
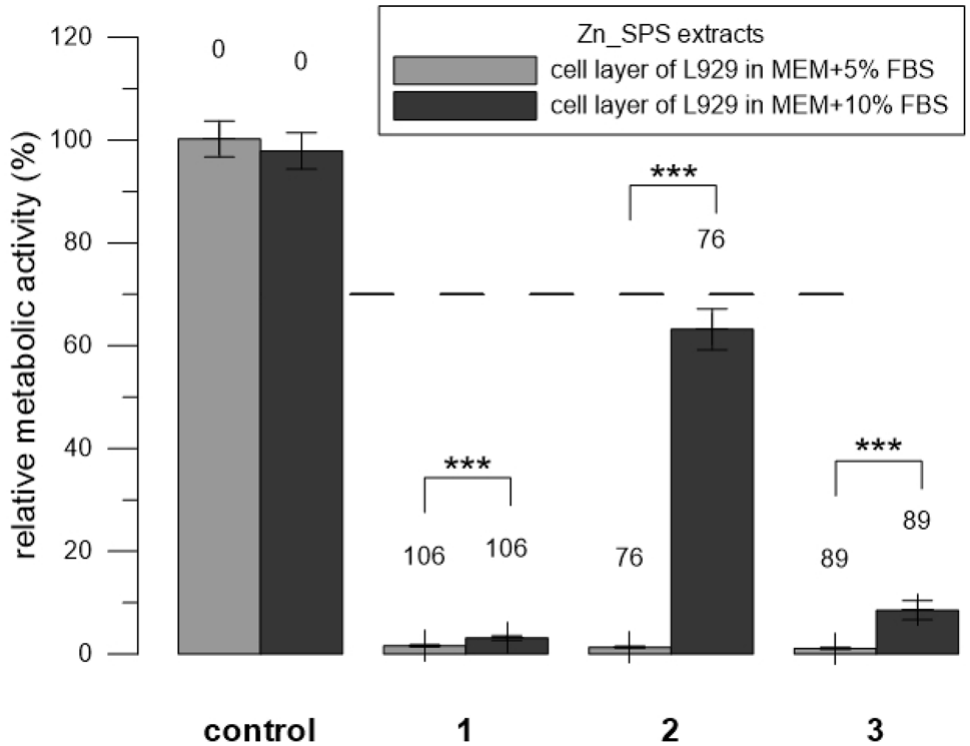
Relative metabolic activity of L929 cells (resazurin assay) after 24 h incubation with extracts of Zn_SPS. Three replicates 1–3 were used. Extracts were prepared in medium either with 5% or with 10% FBS. Sole extraction medium served as a control (unaffected cells which metabolic activity was taken as 100%). Numbers above the columns stand for the concentration of measured Zn in µM. The dashed line stands for the normative limit of 70% metabolic activity of the control. Error bars stand for the standard error deviation of six measurements. Significance codes according to p value: 0 ‘***’0.001 ‘**’0.01 ‘*’0.05 (two-sample t-test).

**Figure 4:**
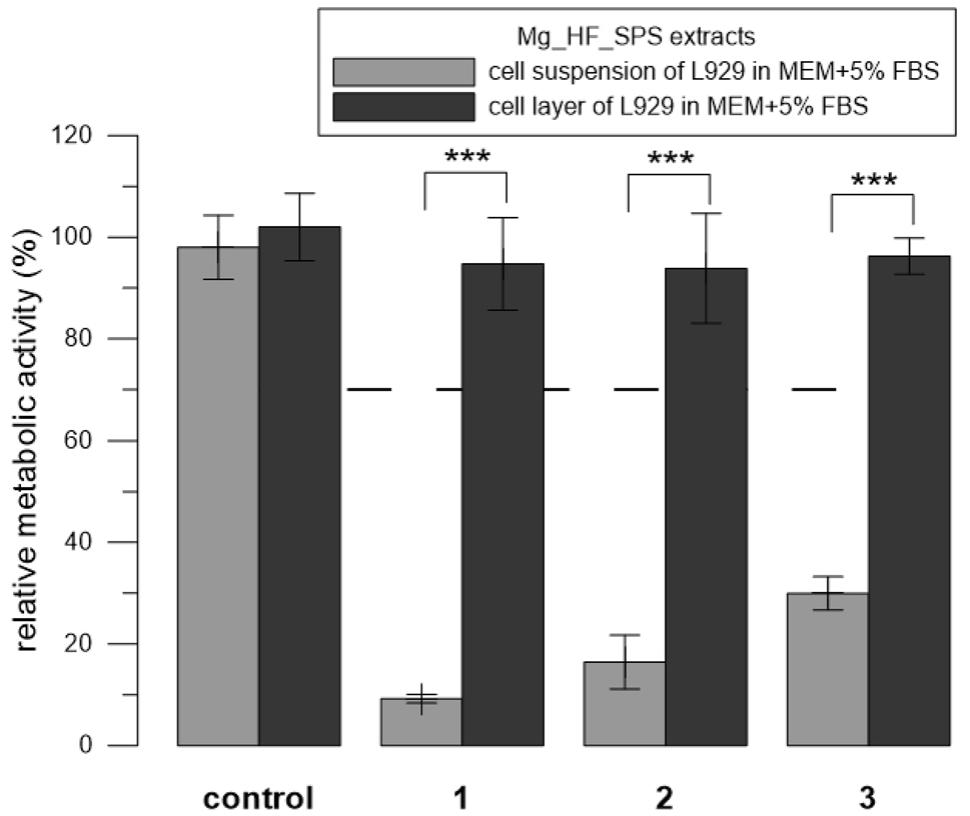
Relative metabolic activity of L929 cells (resazurin assay) after 24 h incubation with extracts of Mg_HF_SPS. Three replicates 1–3 were used. The extract was added either to the cell layer or to the cell suspension. Sole extraction medium served as a control (unaffected cells which metabolic activity was taken as 100%). The dashed line stands for the normative limit of 70% metabolic activity of the control. Error bars stand for the standard error deviation of six measurements. Significance codes according to p value: 0 ‘***’0.001 ‘**’0.01 ‘*’0.05 (two-sample t-test).

**Figure 5:**
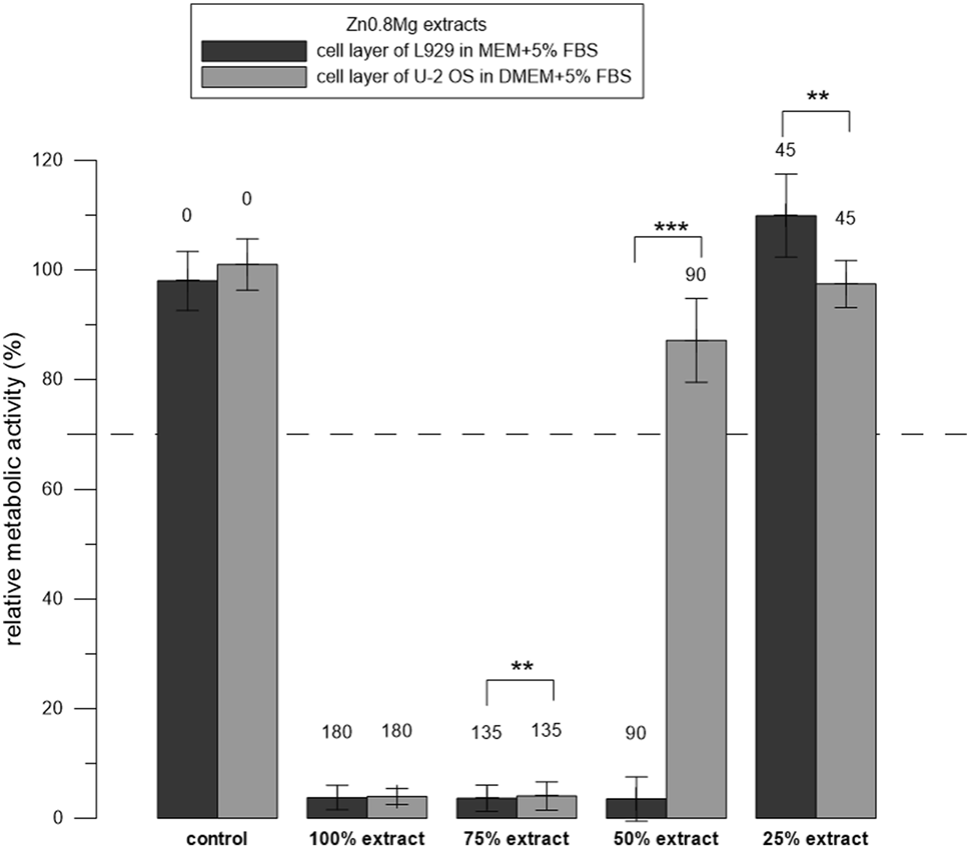
Relative metabolic activity of the cells (resazurin assay) after 24 h incubation with extracts of Zn0.8 Mg. Three replicates 1–3 were used. The extract was added either to L929 or to U-2 OS cells. Sole extraction medium served as a control (unaffected cells which metabolic activity was taken as 100%). Numbers above the columns stand for the concentration of measured Zn in µM. The dashed line stands for the normative limit of 70% metabolic activity of the control. Error bars stand for the standard error deviation of six measurements. Significance codes according to p value: 0 ‘***’0.001 ‘**’0.01 ‘*’0.05 (two-sample t-test).

The statistical analysis was done in R software^18^. Two variances were compared using F test and either two-sample t-test or Welch two-sample t-test was performed. Samples compared are indicated in Figs. 2, 3, 4, and 5.

## Results

### Simulated extracts

We incubated the cells with simulated extracts (ZnCl_2_ solutions) and determined their toxicity at various conditions of the test. Solutions of ZnCl_2_ were significantly (p ˂ 0.001) less toxic in 10% FBS than in 5% (Fig. 2A). Zn concentration of 80 µM was toxic towards L929 cells in medium MEM with 5% FBS whereas it was not toxic towards L929 cells in medium MEM with 10% FBS. Solutions of ZnCl_2_ were significantly (p ˂ 0.001) less toxic in DMEM than in MEM (Fig. 2B).

Zn concentration of 120 µM was toxic towards L929 cells in medium MEM with 10% FBS whereas it was not toxic towards L929 cells in medium DMEM with 10% FBS. Solutions of ZnCl_2_ were significantly (p ˂ 0.001) less toxic when mixed with freshly trypsinized suspension compared to the addition of the solutions to the cell layer (Fig. 2C). Zn concentration of 120 µM was toxic towards L929 cells in medium MEM with 10% FBS when added to the monolayer whereas it was not toxic towards L929 cells in medium MEM with 10% FBS when added to the cell suspension. ZnCl_2_ solutions were significantly (p ˂ 0.001) less toxic to U-2 OS cells than to L929 cells (Fig. 2D). Zn concentration of 120 µM was toxic towards L929 cells in medium MEM with 10% FBS whereas it was not toxic towards U-2 OS cells in medium MEM with 10% FBS.

### Extracts of alloys

To determine the parameters affecting the results of testing the cytotoxicity of alloys, we incubated cells with the extracts and tested the following conditions. When testing Zn_SPS, we used two concentrations of FBS. We performed the extraction in MEM + 5% FBS and after the extraction, we either supplemented it either with MEM + 5% FBS (final concentration of FBS was 5%) or we supplemented it with pure FBS (the final concentration of FBS was 10%). Similarly to the solutions of ZnCl_2_, the extracts of Zn_SPS were significantly (p ˂ 0.001) less toxic in 10% FBS than in 5%. To be more specific, the extracts were toxic in the majority of cases; but the lowest concentration of 76 µM (replicate 2) showed the most pronounced difference between the two concentrations of FBS (Fig. 3). When testing Mg_HF_SPS, we used two ways of addition of the extract to the cells, i.e., to the cell layer and to the cell suspension (Fig. 1). Contrary to the solutions of ZnCl_2_ (Fig. 2), the extracts of Mg_HF_SPS were significantly (p ˂ 0.001) more toxic when mixed with freshly trypsinized suspension compared to the addition of the extracts to the adherent cell layer in case of all three replicates used (Fig. 4). When testing Zn0.8 Mg, we used two types of cells. Similarly to the solutions of ZnCl_2_, diluted extracts of Zn0.8 Mg were significantly (p ˂ 0.01) less toxic for U-2 OS cells than for L929 cells. To be more specific, Zn concentration of 90 µM were not toxic towards U-2 OS cell but were toxic towards L929 cells (Fig. 5).

## Discussion

In vitro experiments represent the first stage of testing of newly developed biomaterials. However, the standard ISO 10993-5 gives the researcher the great freedom to choose conditions of the test. This study aimed to show important factors influencing the results of cytotoxicity tests of extracts from biodegradable metals.

The simplest way to compare different conditions of the test is to use so-called simulated extracts, i.e., solutions of ZnCl_2_ to simulate extracts of zinc alloys. On the contrary, we were not successful in the simulation of extracts of Mg-based materials, where not only the concentration of released ions but also pH had a profound effect (our unpublished data). Figure 2 shows that parameters such as FBS concentration, type of medium, type of exposition, and type of cells all affect the resulting viability (measured as metabolic activity) of the cells exposed to simulated extracts.

Solutions of ZnCl_2_ were less toxic as the concentration of FBS increased (Fig. 2A). As mentioned in the standard ISO 10993-5, FBS has a protective effect since it may bind and thus mask toxic substances including Zn ions. We observed the same effect of FBS in the case of extracts of Zn_SPS (Fig. 3). In this case, we used the same concentration for extraction and then supplemented the extract with additional FBS. The relatively high affinity and abundant protein albumin in FBS probably bound a significant portion of the released zinc^19^ and thus caused a decrease in toxicity.

Next, we compared two types of media. The solutions of ZnCl_2_ were less toxic towards L929 cells in DMEM than in MEM (Fig. 2B). The reason for that may be that DMEM (Sigma, D0819) is richer than MEM (Sigma, M0446). It contains more glucose (25 mM in DMEM vs. 5.6 mM in MEM) and the concentration of amino acids and vitamins in DMEM is also two and fourfold higher, respectively. DMEM also contains additional non-essential amino acids which are missing in MEM. These differences in composition probably improve cell fitness and decrease the cytotoxic effect of the extracts in DMEM compared to MEM medium. Nevertheless, MEM is the medium designated for the cultivation of L929 cells recommended by the ISO standard and moreover, by its Na^+^, bicarbonate and glucose concentration is closer to human plasma (Table 3) and thus, closer to clinical conditions. As can be seen from Fig. 3, the elution of elements from one type of sample can vary. It is known that both Zn-based and Mg-based alloys suffer from irregular corrosion^20–23^. This means that the material is not dissolved homogenously from the whole surface, but some areas may corrode preferably, which is referred as localized corrosion. This is generally connected to the presence of various phases in the microstructure including impurities, which act in most cases as cathodic sites and accelerate corrosion of anode in the form of magnesium or zinc matrix. However, it is worth mentioning that localized corrosion is often observed also on pure metallic materials (Mg, Zn), especially due to even accidental presence of impurities at some locations. Such behaviour may occur randomly depending on the microstructure of the material and immediately affect the concentration of released ions in solution. Therefore, various samples of the same material may release slightly various amounts of ions. Besides this, due to the accelerated corrosion process, a bigger volume of non-protective porous corrosion products is formed at these areas. These products may differ in composition, which at the same time affect the real concentration of ions released to the solution^21,23^. Finally, some differences of released ions in the corrosion environment may be also observed. This applies not only for longer immersion tests but also short exposures like preparation of extracts in our case.

**Table 3:** Ionic composition and glucose concentration of cultivation media in comparison with human plasma.

Fluid	MEM M0446	DMEM D0819	Human plasma
Component	Concentration [mmol l^−1^]		
Cl^−^	126.9	89.8	95–107
Na^+^	143.6	124.5	142
Ca^2+^	1.8	1.8	2.1–2.6
K^+^	5.4	5.4	3.6–5.5
Mg^2+^	0.8	0.8	1–1.5
HPO_4_^2−^	1.0	0.9	0.65–1.45
SO_4_^2−^	0.8	0.8	0.5
HCO_3_^2−^	26.2	44.0	22–30 (27)
Glucose	5.6	25	5.0

We also tested two different ways of addition of the extracts to the cells, i.e., to the sub-confluent cell layer or to the freshly prepared cell suspension (Fig. 1). Solutions of ZnCl_2_ were less toxic when mixed with freshly trypsinized suspension compared to the addition of the solutions to the adhered cell layer (Fig. 2C). The reason for that is probably related to the method of preparation of the cell suspension (see “Cell culture conditions” and “Exposition to the extracts using the cell suspension (Fig. 1, 2nd mode) sections”) and to the major factor causing the toxicity of the extracts. During the suspension preparation, trypsin is used to detach cells growing on the surface of the cultivation plate. After a couple of minutes, trypsin was neutralised by the standard procedure, i.e., by the addition of medium with FBS, there was no centrifugation step. The content of EDTA, a chelating agent, in residual trypsin–EDTA solution probably subsequently chelated Zn ions and prevented their harmful effect on the cells. On the contrary, in the case of the extract of Mg_HF_SPS, the prominent cause of toxic effect were not released ions, but another factor, i.e., elevated pH. We assume that suspension cells were more sensitive to these alkali conditions compared to the adhered cells. The ISO standard allows both modes of exposition. Although the choice dramatically influences the results of cytotoxicity, we have not found any papers comparing those two modes of exposition.

Not surprisingly, the assay was also cell type-dependent. Apart from L929 cells mentioned by the standard as a possible cell model to be used, we also used U-2 OS cells derived from human osteosarcoma, which are one of the model cells (so-called “osteoblast-like cells”) used for in vitro testing of orthopaedic implants^24^. Lower toxicity of ZnCl_2_ solutions towards U-2 OS cells than towards L929 cells (Fig. 2D) was in accordance with the known sensitivity of the L929 cells mentioned in the standard ISO 10993-5. In the case of the extracts of Zn0.8 Mg, there was a combined effect of the cell type as well as the medium type (Fig. 5). Lower sensitivity of osteoblasts (MG-63) to the selected ions in comparison with other cell types was also observed by Feyerabend et al.^25^. Conversely, Yamamoto et al. claimed that the toxicity of the 43 tested salts was independent of the cell type^26^. Differences in observations could be influenced also by other factors such as cell density^27^ or passage number^28^.

## Conclusions

We have conducted a thorough testing of the possible factors influencing the results of the in vitro cytotoxicity testing of metallic biomaterials, which are not specified in the ISO standard. For this, we have used Zn- and Mg-based alloys as well as so-called simulated extracts. The type of medium, the concentration of FBS, mode of exposition, and cell type all influence the cytotoxicity of the extracts. The obvious dilemma whether to simulate clinical conditions or whether to perform the in vitro test at exaggerated conditions to guarantee the safety of the tested material in vivo should be addressed. Based on our results, we strongly recommend not to use only the usual statement “in vitro cytotoxicity was performed according to ISO 10,993 standard”, but to thoroughly describe all the aforementioned conditions used during the cytotoxicity testing including catalogue numbers of media. Collectively, it could make the different studies more comparable.

## References

[1] Wolf MF, Coleman KP, Lewerenz GM, Hoffman AS, Schoen FJ, Lemons JE (2013). Chapter II. 3.3—In vitro assessment of cell and tissue compatibility A2—Ratner, Buddy D. Biomaterials Science.

[2] Jablonská E, Horkavcová D, Rohanová D, Brauer DS (2020). A review of in vitro cell culture testing methods for bioactive glasses and other biomaterials for hard tissue regeneration. J. Mater. Chem. B.

[3] J.M. Anderson, R.W. Bianco, J. Grehan, F., B.C. Grubbs, S.R. Hanson, K.D. Hauch, M. Lahi, J.P. Mrachek, S. Northup, J., Biological Testing of biomaterials, in: B.D. Ratner, A.S. Hoffman, F.J. Schoen, J.E. Lemons (Eds.) Biomaterials science: an introduction to materials in medicine, Elsevier Amsterdam, 2004.

[4] Zheng YF, Gu XN, Witte F (2014). Biodegradable metals. Mater. Sci. Eng. R. Rep..

[5] Fischer J, Profrock D, Hort N, Willumeit R, Feyerabend F (2011). Reprint of: Improved cytotoxicity testing of magnesium materials. Mater. Sci. Eng. B-Adv..

[6] Wang J, Witte F, Xi T, Zheng Y, Yang K, Yang Y, Zhao D, Meng J, Li Y, Li W, Chan K, Qin L (2015). Recommendation for modifying current cytotoxicity testing standards for biodegradable magnesium-based materials. Acta Biomater..

[7] Yao T, Asayama Y (2017). Animal-cell culture media: History, characteristics, and current issues. Reprod. Med. Biol..

[8] Yamamoto A, Hiromoto S (2009). Effect of inorganic salts, amino acids and proteins on the degradation of pure magnesium in vitro. Mater. Sci. Eng. C.

[9] Minarik P, Jablonska E, Kral R, Lipov J, Ruml T, Blawert C, Hadzima B, Chmelik F (2017). Effect of equal channel angular pressing on in vitro degradation of LAE442 magnesium alloy. Mater. Sci. Eng. C Mater. Biol. Appl..

[10] Kirkland N, Birbilis N (2014). Introduction to Magnesium Biomaterials.

[11] Liu CL, Wang YJ, Zeng RC, Zhang XM, Huang WJ, Chu PK (2010). In vitro corrosion degradation behaviour of Mg-Ca alloy in the presence of albumin. Corros. Sci..

[12] Li P, Schille C, Schweizer E, Kimmerle-Müller E, Rupp F, Heiss A, Legner C, Klotz UE, Geis-Gerstorfer J, Scheideler L (2019). Selection of extraction medium influences cytotoxicity of zinc and its alloys. Acta Biomater..

[13] Shah FA, Brauer DS, Wilson RM, Hill RG, Hing KA (2014). Influence of cell culture medium composition on in vitro dissolution behavior of a fluoride-containing bioactive glass. J. Biomed. Mater. Res. A.

[14] Dvorsky D, Kubasek J, Vojtech D (2018). A new approach in the preparation of biodegradable Mg-MgF2 composites with tailored corrosion and mechanical properties by powder metallurgy. Mater. Lett..

[15] Čapek J, Pinc J, Msallamová Š, Jablonská E, Veřtát P, Kubásek J, Vojtěch D (2019). Thermal plasma spraying as a new approach for preparation of zinc biodegradable scaffolds: A complex material characterization. J. Therm. Spray Technol..

[16] Kubasek J, Vojtech D, Jablonska E, Pospisilova I, Lipov J, Ruml T (2016). Structure, mechanical characteristics and in vitro degradation, cytotoxicity, genotoxicity and mutagenicity of novel biodegradable Zn-Mg alloys. Mater. Sci. Eng. C Mater. Biol. Appl..

[17] Riss TL, Moravec RA, Niles AL, Duellman S, Benink HA, Worzella TJ, Minor L, Sittampalam GS, Coussens NP (2016). Cell viability assays. Assay Guidance Manual.

[18] R Core Team. R: A language and environment for statistical computing, R Foundation for Statistical Computing, Vienna, Austria (2020).

[19] Bozym RA, Chimienti F, Giblin LJ, Gross GW, Korichneva I, Li Y, Libert S, Maret W, Parviz M, Frederickson CJ, Thompson RB (2010). Free zinc ions outside a narrow concentration range are toxic to a variety of cells in vitro. Exp. Biol. Med. (Maywood).

[20] Ding Y, Wen C, Hodgson P, Li Y (2014). Effects of alloying elements on the corrosion behavior and biocompatibility of biodegradable magnesium alloys: A review. J. Mater. Chem. B.

[21] Atrens A, Song G-L, Liu M, Shi Z, Cao F, Dargusch MS (2015). Review of recent developments in the field of magnesium corrosion. Adv. Eng. Mater..

[22] Atrens A, Shi Z, Mehreen SU, Johnston S, Song G-L, Chen X, Pan F (2020). Review of Mg alloy corrosion rates. J. Magnesium Alloys.

[23] Venezuela J, Dargusch MS (2019). The influence of alloying and fabrication techniques on the mechanical properties, biodegradability and biocompatibility of zinc: A comprehensive review. Acta Biomater..

[24] Saldana L, Bensiamar F, Bore A, Vilaboa N (2011). In search of representative models of human bone-forming cells for cytocompatibility studies. Acta Biomater..

[25] Feyerabend F, Fischer J, Holtz J, Witte F, Willumeit R, Drucker H, Vogt C, Hort N (2010). Evaluation of short-term effects of rare earth and other elements used in magnesium alloys on primary cells and cell lines. Acta Biomater..

[26] Yamamoto A, Honma R, Sumita M (1998). Cytotoxicity evaluation of 43 metal salts using murine fibroblasts and osteoblastic cells. J. Biomed. Mater. Res..

[27] Wataha JC, Hanks CT, Craig RG (1993). The effect of cell monolayer density on the cytotoxicity of metal-ions which are released from dental alloys. Dent. Mater..

[28] Wataha JC, Hanks CT, Sun ZL (1994). Effect of cell-line on in-vitro metal-ion cytotoxicity. Dent. Mater..

